# The Orientation Selectivity of Spike-LFP Synchronization in Macaque V1 and V4

**DOI:** 10.3389/fncom.2019.00047

**Published:** 2019-07-11

**Authors:** Zhaohui Li, Mengyu Gao, Yongtian Wang

**Affiliations:** ^1^School of Information Science and Engineering, Yanshan University, Qinhuangdao, China; ^2^Hebei Key Laboratory of Information Transmission and Signal Processing, Yanshan University, Qinhuangdao, China

**Keywords:** orientation selectivity, multi-electrode array, spike, local field potential, synchronization

## Abstract

Orientation selectivity is a fundamental property of visual cortical neurons and plays a crucial role in pattern perception. Although many studies have dedicated to explain how the orientation selectivity emerged, the mechanism underlying orientation selectivity is still not clear. In this work, we investigated the synchronization between spikes and local field potentials (LFP) in gamma band, with the aim of providing a new avenue to analyze the orientation selectivity. The experimental data were recorded by utilizing two chronically implanted multi-electrode arrays, where each array consisted of 48 electrodes and was placed over V1 and V4, respectively, in two macaques performing a selective visual attention task. An unbiased and robust measure for quantifying the synchronization between spikes and LFP was employed in the analysis process, which is termed as spike-triggered correlation matrix synchronization (SCMS) and performs reliably for limited samples of data. We observed the spike-LFP synchronization in three cases, i.e., spikes and LFP in V1, spikes and LFP in V4, spikes in V4 and LFP in V1. From the orientation tuning curves based on the spike-LFP synchronization, it is found that there is a strong correlation between the synchronization and grating orientation. The neurons in both V1 and V4 exhibit orientation selectivity, but V1 is stronger. In addition, the spike-LFP synchronization strength between V1 and V4 also shows orientation selectivity to drifting gratings. It means that the synchronization not only reflects the basic features of visual stimulation, but also describes the orientation tuning characteristics of neurons in different regions. Our results suggest that the spike-LFP synchronization can be used as an alternative and effective method to study the mechanism for generating orientation selectivity of visual neurons.

## Introduction

The orientation is a basic feature of natural images. The orientation selective response of visual cortical neurons to the object boundary plays a key role in the shape perception and other perception processes (Hubel and Wiesel, 1962; Mansfield, 1974; Girshick et al., 2011; Durant et al., 2017). In the past decades, many studies have been devoted to explore the mechanism for generating orientation selectivity. Generally, two important types of signal were employed in these analyses, i.e., spikes (action potentials) and local field potentials (LFP), which were simultaneously recorded from the visual cortex by multiple electrode arrays (Zhang and Li, 2013; Bharmauria et al., 2016). On the one hand, the spikes are identified by high-pass filtering, detection and sorting, indicating the firing activities of individual neurons. Its firing rate has been widely used since the orientation selectivity is discovered, e.g., some researchers found that the spike firing rate has different response values under different orientation and contrast stimuli (Hubel and Wiesel, 1962; Anderson et al., 2000; McLaughlin et al., 2000; Manyakov and Van Hulle, 2010). It indicates that the neuronal discharge activity is able to encode the orientation information of visual images. On the other hand, the LFP is obtained by low-pass filtering the original wideband signal, representing the synaptic activities of local populations of cortical neurons (Buzsaki et al., 2012; Gaucher et al., 2012). Because the spike firing rate cannot reflect the synaptic activities of multiple neurons in a local region, the LFP frequency or energy is adopted in many studies. For example, the high frequency oscillations of LFP in the striate cortex of awake monkeys showed stronger orientation selectivity than low frequency oscillations (Frien et al., 2000), and the energy variation of the LFP in gamma band was able to effectively encode the stimuli (such as time, frequency, orientation, etc.) in images (Siegel and Konig, 2003; Henrie and Shapley, 2005; Ince et al., 2012).

In recent years, a large number of neurophysiological studies have shown that there is a close relationship between spike and LFP gamma band (Ray and Maunsell, 2011a,b; Li et al., 2014). Combining these two signals to decode the behaviors can provide more information than using one signal separately (Mehring et al., 2003; Mollazadeh et al., 2009), which means that, it is able to provide a comprehensive description about the neural mechanism of signal processing. Moreover, it has been shown that the spike and LFP both participate in the coding of visual information (Quian Quiroga and Panzeri, 2009; Perge et al., 2014). Thus, we think relating the spike-LFP correlation and orientation is an effective tool to investigate the orientation selectivity. Therefore, we used the SCMS method (Li et al., 2016) to estimate the spike-LFP synchronization of the data which was obtained by simultaneously implanting two multi-electrode arrays in V1 and V4 of visual cortex, respectively.

## Materials and Methods

### Experiment Procedure

The experimental data was recorded from two male rhesus monkeys. All procedures were conducted in compliance with the National Institutes of Health Guide for the Care and Use of Laboratory Animals, and were approved by the Institutional Animal Care and Use Committee of Beijing Normal University. Under general anesthesia induced with ketamine (10 mg/kg) and maintained with isoflurane (1.5–2.0%), a titanium post was attached to the skull with bone screws for immobilizing the animal's head during behavioral training. After the monkeys had been trained in a simple fixation task, two 6 × 8 multi-electrode arrays (with interelectrode spacing of 0.4 mm, electrode length of 0.5–0.6 mm, and typical electrode impedances of a few hundred kiloohms; Blackrock Microsystems) were implanted intoV1 and V4, respectively. LFP and spike data were recorded at 10 kHz using a 128-channel Cerebus neural electrophysiological signal recording system (Blackrock Microsystems).

### Visual Stimulation

**Figure 1:**
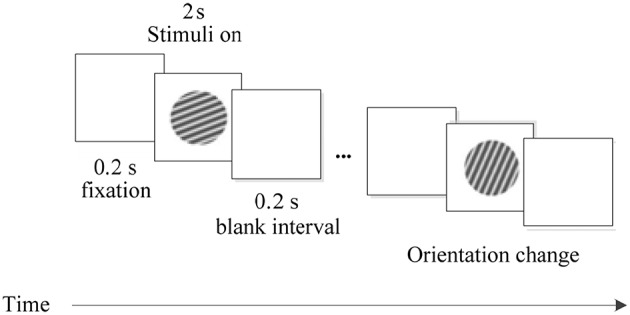
The experimental procedure. Each trial was performed for 2.4 s, including three phases: fixation in the first 0.2 s, stimulus in the next 2 s, and a blank interval in the final 0.2 s.

The visual stimulation in the experiment were generated by a stimulus generator system ViSaGe and displayed on a 22-inch CRT monitor at a viewing distance of 100 cm. The stimulus patterns were drifting sinusoidal gratings of different orientations which were displayed within a circular patch of 4° visual angle in diameter, covering the visual field locations of all recording sites. The orientation of the sinusoidal grating used in the experiment was uniformly distributed between 0° and 360° in steps of 22.5°. Other stimulus settings were identical in the whole experiment, including the contrast of 99%, the spatial frequency of 2 cycle/degree and the temporal frequency of 4 Hz. The experimental procedure is shown in Figure 1.

On each trial, the grating with different orientations was appeared on the screen in a pseudorandom order. Every stimulus was presented for 2 s and repeated 30 times. A trial started when a lever was pulled by the animal. Then, a fixation point (FP) of 0.1° was displayed in the CRT center. Within 600 ms after FP presentation, the animal was required to fixate within an invisible circular window of 0.6° in radius around the FP. Before the stimulus was displayed, the animal maintained its fixation on the screen for 200 ms. And after the stimulus, there was a blank interval of 200 ms. The FP was then slightly dimmed, and the animal had to release the lever within 600 ms for a drop of juice as reward.

### Signal Preprocessing

With increasing popularity of LFP analysis, oscillations in LFP gamma band have been used to study orientation selectivity (Berens et al., 2008; Xing et al., 2012). In order to obtain LFP gamma band signal and preserve the phase relationship between LFP and spikes, we used a two-way least-squares FIR filter in the EEGLAB toolbox (Delorme and Makeig, 2004) to perform zero-phase shift band-pass filtering of 30–80 Hz on the original signals recorded in the experiment.

**Figure 2:**
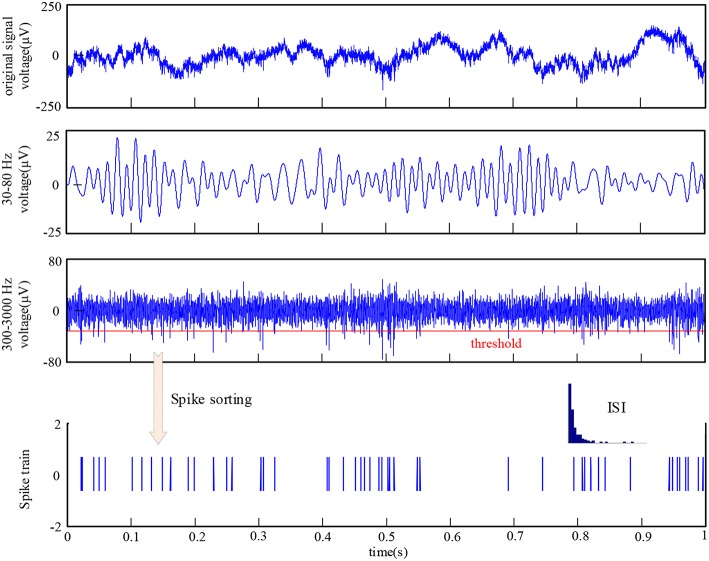
The raw data recorded by one electrode and the procedure of signal preprocessing.

To identify spikes fired by neurons, the recorded signals were first filtered with a band-pass filter of 300–3,000 Hz. Then, the threshold detection method was used to determine the spiking time and extract the spike waveform. Finally, spikes were classified by utilizing an unsupervised detection and sorting method based on wavelets and superparamagnetic clustering (Quiroga et al., 2004). An example of the raw data and the corresponding procedure of signal preprocessing is illustrated in Figure 2. During the implementation of spike sorting, the cluster with most spikes was taken as the firing activity of a neuron recorded by one electrode and the other spikes were discarded. Thus, there were 48 neurons in V1 and V4, respectively. The inter-spike interval (ISI) histogram metric was used to evaluate the spike sorting accuracy. An example of the ISI is shown in bottom of Figure 2. Considering the effect of refractory period, it is an acceptable result of spike sorting for this electrode. The ISI also exhibits similar distribution for other electrodes. The single-unit activity and LFP recorded from the same electrodes were used to calculate the spike-LFP synchronization in this paper.

### Synchronization Analysis

**Figure 3:**
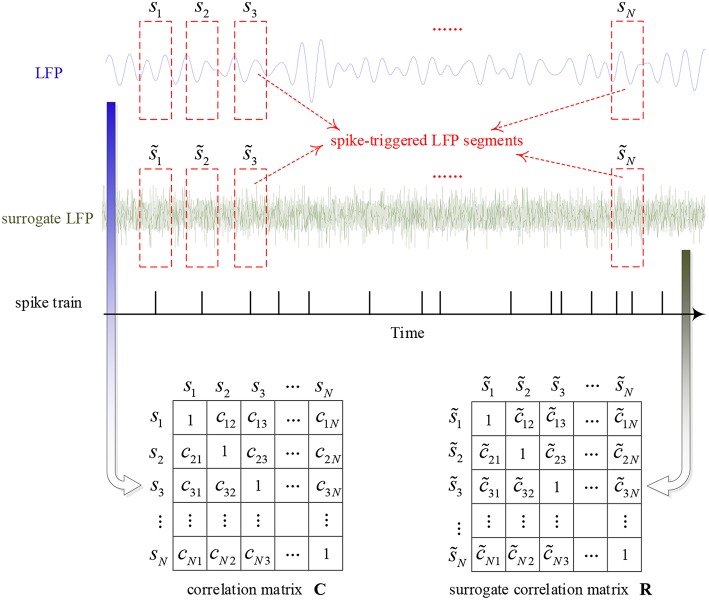
The calculation procedure of the algorithm for characterizing the strength of spike-LFP synchronization.

The SCMS method was used to analyze the data recorded in the macaques' visual cortex V1 and V4. The main idea of this method is to take the LFP segments centered on each spike as multi-channel signals and measure the synchronization between these LFP signal segments using the phase locking value. The global synchronization is calculated by constructing a correlation matrix to quantify the coupling strength of the spike and LFP. In the data analysis, the influence of window length on the algorithm is very small. However, it is possible that there are other spikes immediately before or after a specific spike which may alter the frequency and phase of the LFP (Zanos et al., 2011). On the other hand, the algorithm uses the similarity of variation in LFP phase as the mechanism for the calculation of spike-LFP synchronization. Considering the impact of these two aspects, we used a window of 20 ms in this study. More details are as follows and the calculation procedure is shown in Figure 3.

First, the instantaneous phase of the whole filtered LFP signal is calculated by Hilbert transform. Then, construct the correlation matrix **C** by calculating the phase locking value between pairs of LFP segments, i.e.,

(1) $$\begin{array}{r} c_{mn}=\left|\frac{1}{M}\overset{M}{\underset{k=1}{\sum }}{\text{e}}^{i\left(\phi_{m}\left(t_{k}\right)-\phi_{n}\left(t_{k}\right)\right)}\right| \end{array}$$

where ϕ_*m*_(*t*_*k*_) and ϕ_*n*_(*t*_*k*_) denotes the phase of mth and nth LFP segments, respectively, *t*_*k*_ is the sampling time and *M* denotes the number of samples in the time window. All elements of matrix **C** range from 0 to 1: when *c*_*mn*_ = 1, there is a perfect phase synchronization between the mth and nth LFP segments; and when *c*_*mn*_ = 0, there is no synchronization. Thus, **C** is a real symmetric matrix of order *N* and all diagonal elements are equal to 1, where *N* denotes the number of LFP segments. Moreover, the eigenvalues of matrix **C** (λ_1_ ≥ λ_2_ ≥ ⋯ ≥ λ_*N*_) are real numbers and the sum of them is *N*. If all of the LFP segments are totally non-synchronized with each other, **C** will become an identity matrix and all of the eigenvalues will be equal to 1. Once all of the LFP segments are perfectly synchronized, the maximum eigenvalue of **C** will be equal to *N* and other eigenvalues zero. Above all, eigenvalues can provide information about the synchronization between LFP segments.

### Surrogate Data

Finally, in order to obtain a normalized value of spike-LFP synchronization which is independent of the number of spikes, this paper used the Rank-Shuffled Surrogate (RSS) method to generate surrogate data (Junfeng et al., 2012). Assume that {g(n)} denotes a Gaussian random sequence, and *R*[g(k)] denotes the order in which g(k) is ranked in the time series {g(n)}. For example, if g(k) is the 5th smallest sample point in {g(n)}, then *R*[g(k)] = 5. Then, use $\tilde{s}\left(\text{n}\right)$ to represent the rank-shuffled surrogate data of the original signals {s(n)}, where $\tilde{s}\left(\text{n}\right)$ = s [ k(n) ], and k(n) = R [ g(n) ]. That is to say, the surrogate data is generated by randomizing the order of the original signals, destroying the time structure, but retaining the amplitude distribution, mean and variance.

By using such a method, all spike-triggered LFP segments are randomized to calculate a surrogate correlation matrix **R**. That is, the surrogate data is generated by randomizing the order of the original signals. Similarly, the ordered eigenvalues of surrogate correlation matrix **R** can be obtained, which are denoted as ${\lambda_{1}}^{s}\geq {\lambda_{2}}^{s}\geq \cdots \geq {\lambda_{N}}^{s}$. This randomization process is repeated and calculated 100 times, the mean and standard deviation of the maximum eigenvalues are denoted as $\bar{{\lambda_{1}}^{s}}$and σ_1_, respectively. Then, the normalized spike-LFP synchronization value is calculated by the following equation:

(2) $$\eta =\{\begin{array}{c} \left(\frac{\lambda_{1}-\bar{\lambda_{1}{}^{s}}}{N-\bar{\lambda_{1}{}^{s}}}\right)\;if\;\lambda_{1}>\left(\bar{\lambda_{1}{}^{s}}+K\times \sigma_{1}\right)\\ 0\text{ otherwise} \end{array}$$

where *K* is a constant that determines the threshold, and *K* = 3 is selected for 99% confidence intervals (Li et al., 2007).

### Circle Variance

The circle variance (CV) (Ringach et al., 2002) is an orientation selectivity index obtained by the vector sum of neuron's responses to each orientation of the stimulus divided by the scalar sum of the responses, which can effectively describe the degree of orientation selectivity. Its definition is:

(3) $$\begin{array}{r} \text{CV}=1-\frac{\underset{k}{\sum }r_{k}\exp\left(\text{i2}{\;\theta \;}_{k}\right)}{\underset{k}{\sum }r_{k}} \end{array}$$

## Results

In order to acquire more accurate and significant results, the trials with very few spikes (<10) and distorted recordings with very small amplitude are rejected, and then the mean spike-LFP synchronization of the remaining trials is calculated. The experimental data was analyzed using Matlab.

### Orientation Selectivity of Neurons in V1 and V4

**Figure 4:**
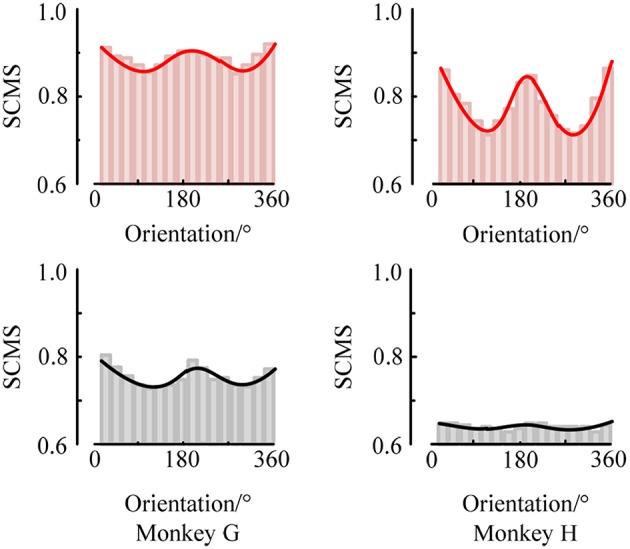
The mean spike-LFP synchronization values in V1 and V4 of the two monkeys. Response of the V1 neurons is indicated by a red line, and response of the V4 neurons is indicated by a black line.

First, to examine the neuronal response in the two brain regions V1 and V4 under the stimuli of sinusoidal grating with different orientations, we used the SCMS method to estimate the spike-LFP synchronization of the experimental data recorded by each electrode respectively. The mean spike-LFP synchronization values of the 48 electrodes in V1 and V4 are plotted in the curve of Figure 4.

The red curves show the response of neurons in V1 for the two monkeys. It can be seen that the mean spike-LFP synchronization values exhibit obvious orientation selectivity. More concretely, the neurons respond more strongly to the gratings around 22.5° or 202.5° than the other orientations, which means that the synchronization values to the preference-oriented stimulus and the non-preferential orientation stimulus are markedly different. And the values distribute symmetrically. On the other hand, the black curves show the response of neurons in V4. Clearly, although the distribution of the mean spike-LFP synchronization values is almost symmetric, the difference between them is not as obvious as V1. Especially for monkey H, the neurons did not respond well to the stimulus. There are two reasons may lead to this phenomenon. One is that there are individual differences in the two monkeys and they responded not consistently to the drift gratings. Another, and more important, is that the V4 cortex itself has small patches that encode shape and orientation (Roe et al., 2012). In the experiment, the electrodes in V4 of Monkey H were more likely located close to the color-coded region.

Moreover, the synchronization value of the neurons in V1 is higher than the synchronization value of neurons in V4, indicating that the neurons in V1 are more active and more sensitive to the orientation of the drift grating. Considering that the local field potential is the sum of the excitatory and inhibitory postsynaptic potentials in the vicinity of the recording electrode, it is the superposition of the neuron cluster firing activity in a local area. Then, the synchronization relationship between the spike train fired by a neuron and the local field potential can be understood as the connection between a single neuron and multiple neurons around it. Therefore, it can be considered that the activity of a single neuron in V1 is more affected by the network composed of peripheral neurons, while the neurons in V4 are relatively less affected by peripheral neurons.

**Figure 5:**
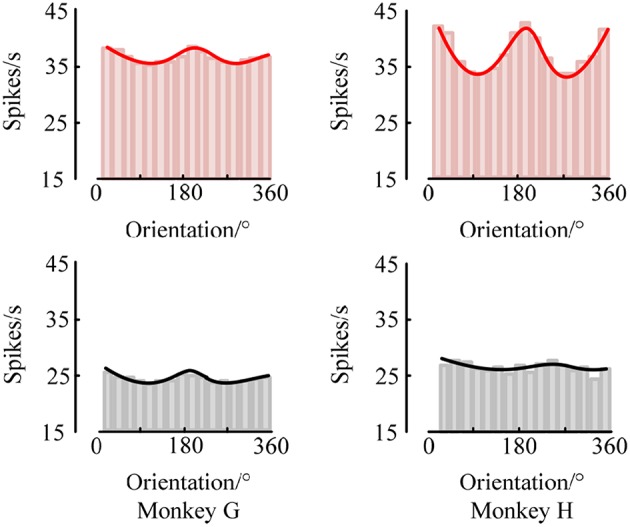
The orientation tuning of the spiking response. The values represent the mean firing rates of neurons to sinusoidal grating with different orientations.

At the same time, we also analyzed the orientation tuning of the spiking response under different orientations toward grating stimulation. It can be easily seen from Figures 4, 5 that in the two brain regions V1 and V4 under the grating stimulation, peaks appear in the orientation tuning curves based on both the firing rate and the spike-LFP synchronization. This indicates that both the spiking response and the spike-LFP synchronization of neurons have a clear orientation selectivity. Meanwhile, it can be found that the firing rate of neurons in the two brain regions is different. The mean firing rate of neurons in V1 is higher than that in V4, indicating that the neurons in V1 are active and have more firing activity under grating stimulation. Similar results were observed for the mean intensity of the spike-LFP synchronization.

**Figure 6:**
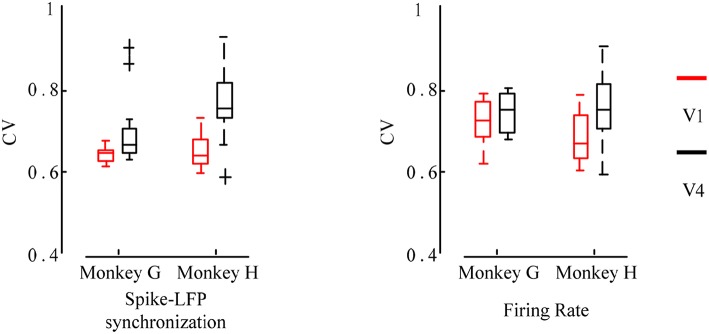
Box plot of the CV values for the firing rate and the spike-LFP synchronization of neurons in different regions. Red indicates the V1 and black indicates the V4. The smaller the CV values, the more significant the orientation selectivity of the neurons.

Second, we used the CV to measure the orientation selectivity of neurons and compared the differences between different brain regions. The CV values were calculated separately for the orientation tuning curves obtained by the two methods of the firing rate and the spike-LFP synchronization, and then the neurons in the two brain regions V1 and V4 were statistically analyzed. The result is shown in Figure 6.

It can be seen that the CV values obtained by the spike-LFP synchronization are smaller than the CV values obtained by the firing rate. All neurons have significant orientation selectivity and most of the CV values range from 0.6 to 0.8. In addition, it can also be found that the CV values of the neurons in V1 is lower than that in V4. The *F* test revealed a significant difference between these two regions (*p* < 0.05). It further indicates that the neurons in V1 exhibit stronger orientation selectivity than that in V4. This shows that for sinusoidal grating with different orientations, the firing activity of neurons will show a certain orientation selectivity. However, this orientation selectivity is stronger when considering the spike-LFP synchronization, that is, the synchronization relationship is more sensitive to grating stimulation with different orientations. Therefore, it can be considered that studying the spike-LFP synchronization relationship provides a more effective method for exploring the formation mechanism of visual neurons toward orientation selectivity. It is able to effectively describe the orientation tuning characteristics of neurons and the difference of orientation selectivity in different regions.

### Co-modulation Effect on Orientation in V1 and V4

**Figure 7:**
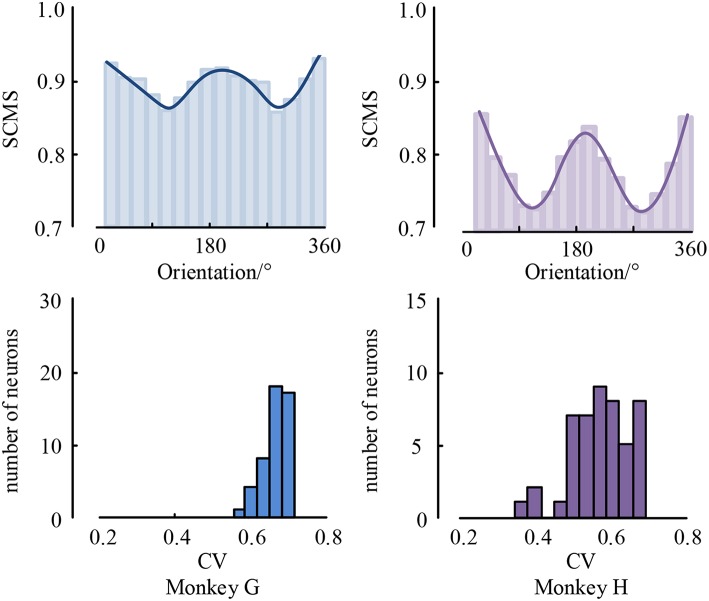
The mean spike-LFP synchronization values between V1 and V4 and the histogram of CV values.

As is known, the processing of visual information requires mutual communication and cooperation among multiple brain areas (Jensen and Mazaheri, 2010; Akam and Kullmann, 2014; Fries, 2015). However, it is still elusive whether and how the distant cortical areas cooperate in visual tasks (Tiesinga and Buia, 2009; Ter Wal and Tiesinga, 2017). For example, whether LFPs coordinate spiking output timely between distant cortical areas that have been traditionally associated with the sensory encoding of visual information, and is the precision of coordination between these areas related to changes in visual information? To understand these questions, we used local field potential and spike recorded in monkeys performing a visual task to study neural interactions between visual area V1 and V4. The result is shown in Figure 7.

As can be seen, the spike-LFP synchronization between V1 and V4 is modulated by visual information content, and its intensity also shows orientation selectivity to drifting gratings during the stimulation. Moreover, all neurons have significant orientation selectivity with CV values less than 0.8. In addition, we also found that the spike-LFP synchronization value between regions is higher than in a single region. This suggests that the spike-LFP synchronization coordinates potential communication between V1 and V4. Specifically, the spike-LFP synchronization is enhanced during visual tasks in both V1 and V4, and increased synchronization is accompanied by the phase coding of visual stimulus.

Moreover, spiking activity in V4 was more strongly locked to LFP in V1 and vice versa, i.e., V4 spiking seems to be more sensitive to V1 gamma than V1 spiking to V4 gamma. The asymmetry of spike-LFP synchronization between the regions implies a possible directedness in the interaction and communication pattern between the regions, the details of which remain to be explored.

## Discussion

We combined spike and LFP signals to investigate the orientation tuning characteristics of neurons in macaques' V1 and V4 under drifting sinusoidal gratings by calculating the synchronization between spike and LFP gamma band. The results are as follows:

First, we found a strong correlation between the spike-LFP synchronization and the stimulus orientation, which is modulated by the orientation and reflects the basic feature information of the visual stimulation. Second, we also investigated the modulation of orientation selectivity through the spike-LFP synchronization of V1 and V4 neurons. The results show that the spike-LFP synchronization not only can effectively encode the stimulus information for different orientations, but also can distinctly distinguish the orientation tuning characteristics of neurons in different regions. Finally, it was observed that there is a clear mutual modulation of orientation between V1 and V4, suggesting that the neural interaction based on the spike-LFP synchronization between these two long-range cortical regions is related to the coding of visual information.

Our findings are consistent with previous studies. For instance, Frien et al. found that gamma-band LFP displays sharper orientation tuning than slower components of the same recordings in striate cortex of the awake monkey (Frien et al., 2000). Lashgari et al. systematically compared the stimulus selectivity of LFP and neighboring single-unit activity recorded in V1 of awake rhesus monkeys. They demonstrated that LFP and single-unit activity have similar stimulus preferences for orientation, direction of motion, contrast and other features (Lashgari et al., 2012). Womelsdorf et al. determined for each spike its phase relative to the gamma cycle and used the pairwise phase consistency to quantify the concentration of phases around the mean gamma phase. They observed that orientation selectivity is modulated by gamma phase and the spike firing rate that occurred close to a neuron's mean gamma phase is most orientation selective (Womelsdorf et al., 2012). Although these results are closely related to ours in this paper, there are two clear distinctions between them. One is that we used an unbiased and robust measure to quantify the spike-LFP synchronization, which provided a reliable comparison between trials with different spike numbers. Then, it is feasible to investigate the tuning characteristics of spike-LFP synchronization under stimulus with different orientations. Another is that we analyzed the spike-LFP synchronization not limited to V1, but expanded it to V4. And we also studied the mutual modulation between V1 and V4.

Taken together, our results illustrate that the connection between spike-LFP synchronization and orientation not only exists in an individual region (V1 or V4), but also between distant cortical regions (V1 and V4). That is, the neural interaction based on spike-LFP synchronization may be related to the maintenance and communication of information during visual information processing. We suggest that this method provides a new direction to study the formation mechanism of orientation selectivity.

## Ethics Statement

All procedures were conducted in compliance with the National Institutes of Health Guide for the Care and Use of Laboratory Animals, and were approved by the Institutional Animal Care and Use Committee of Beijing Normal University.

## Author Contributions

ZL contributed to simulation design and manuscript preparation. MG contributed to performing simulations and analysis. YW contributed to manuscript preparation.

### Conflict of Interest Statement

The authors declare that the research was conducted in the absence of any commercial or financial relationships that could be construed as a potential conflict of interest.
